# Progress in State Medicine

**Published:** 1897-09-25

**Authors:** 


### PROGRESS IN STATE MEDICINE.

*(Continued from page 306.)*

**Milk** (*continued*).—Hope[1] submitted 168 samples of milk taken within the city of Liverpool to bacteriological examination, with a view to ascertaining the presence or otherwise of the tubercle bacillus. The result obtained was exceedingly instructive, and shows that "2·8 per cent. of the 144 samples of milk taken from sources within the city, and under control of the Health Department, contained the tubercle bacillus, but no less than 29·1 per cent. of 24 samples of milk taken at the railway station on arrival from country places contained the tubercle bacillus. . . . The inference to be drawn from this is strikingly conclusive, viz., that the supervision exercised in the city has proved more effectual than that which obtains in the country, and it is also made clear by the results of this examination that consumers are safer, so far as tuberculosis is concerned, with milk from city shippons than with milk sent in from the country." The milk was tested by injecting a small quantity beneath the skin of various animals, tuberculosis being produced if the bacilli were present in sufficient numbers. The probability of finding tubercle bacilli in large quantities of milk by microscopic measures was found to be extremely small. Bardet[2] emphasises the fact that milk is really a manufactured article, its quality dependent upon the nature of the food and the amount of care bestowed upon the cow. After many analyses he has come to regard "good country milk" as a myth and a delusion, for the increased skill, care, and enforced cleanliness of the town cowkeeper results in the production of a milk very much richer and purer than that sent into our cities from the neglected and ill-regulated byres of rural districts. Town milk is either very good or very poor; in the country there is a uniformly poor standard. The regulations suggested are much similar to those advocated for this country. The pasteurisation of milk by rapidly raising it to a temperature of 68-80 deg. C., and then cooling to 15 deg., is recommended as an excellent means of preserving it for short periods, without loss of flavour. Sterilisation is only accomplished at a temperature of 108-110 deg., the vessels being sealed whilst the milk is still boiling. The flasks should be shaken before being opened to secure a perfect emulsion. The methods devised by Vigier and Gaertner for humanising milk are both equally efficacious. It is worthy of note that Lübert[3] found that peptonised milk kept for 24 hours at 37 deg. C. develops poisonous toxins, giving rise to serious symptoms. The qualities of these toxins are not altered by boiling. Peptonised milk should therefore be made at short intervals in small quantities. Similarly, Vaughan and Perkins[4] describe a toxin present in ice-cream and cheese which have at different places caused epidemics of diarrhœa, vomiting, and collapse. The toxic character of the substance was not destroyed by prolonged heat or boiling. The organism forming the toxin could be differentiated from B. Coli., survived freezing for 29 days, and resisted repeated freezing and thawing. It was found pathogenic for ordinary animals. Harris[5], investigating the conditions under which ice-creams are manufactured, finds that as a rule they come from the poorest and least sanitary portion of the city, where they are made amidst filthy surroundings by workmen who have no idea of cleanliness. The materials used—milk, cornflour, essences, &c.—were in a very dirty condition. In the process of manufacture water from the ice employed in the freezing process gains access to the inside of the vessel and thus may be a source of contamination, for it was shown that it was frequently taken from filthy localities, in the case referred to from the Regent's Canal and from a pond, the water of which showed a high degree of organic pollution. Bacteriological cultures from ice-creams yielded an exceedingly large number of colonies, and amongst the various organisms were Bac. Coli, Proteus vulgaris, Fluorescens, and Streptococci.

**Water.**—The *Lancet* Special Commission on the Metropolitan Water Supply[6] is of much value in view of recent reports by the London County Council. A short history of the London water supply of the past is given, the Metropolitan areas supplied by the various water companies are defined, and a description of each area of distribution given. Each company is then treated individually; the source of the water, nature of storage and filtration, length of pipes, percentage of houses under constant supply, average daily supply, and other details being furnished. The data provided show the gross inequalities which exist between the various parts of London both with regard to the quality and quantity of water supplied, and also in the facilities offered to the consumer for obtaining such supplies.

Thresh[7] notes that rural water supplies are, in spite of recent legislation, still drawn from unsatisfactory sources—shallow wells, polluted streams, unprotected springs, or dirty ponds or ditches. Rural authorities have not availed themselves of the powers conferred by the Public Health Act, 1875, to provide fitting supplies, being deterred by fear of expense, by ignorance of the necessity of such provision, or by want of competent skilled advice. Section 62 of the above Act, and Section 3 of the Public Health Water Act, 1878, state that if a supply can be obtained at a reasonable cost the sanitary authorities may cause the owner to execute the necessary work; at the same time the authority is not relieved of its responsibility to provide a public supply, and a person against whom proceedings are taken may urge that it is the duty of the sanitary authority to provide a public supply. So indefinite and elastic are the terms of these sections that but little use has been made of them. A tank to collect the rain-water from the roof is generally regarded as sufficient means of supply for new houses, although as a rule it will be found to be utterly deficient in quantity and abominable in quality. The public wells provided are generally faultily constructed, and inadequately protected from contamination. Dr. Thresh indicates that the remedy for the parish lies in an appeal to the County Council, who under the recent Parish Councils Act may compel the District Council to make the necessary provision, or may undertake the work themselves. The cost is not necessarily high, and the benefit is speedily appreciated.

Collingridge[8] discusses the subject of ship's water supply with special reference to source, storage, and filtration. To prevent contamination by dirty casks, &c., water should be taken directly from the mains by flexible pipes. With good condensers and careful aëration a palatable manufactured water can be produced, and inasmuch as it is free from specific contamination this method is preferable to storage and filtration. Storage tanks should be of iron coated with cement wash. Each tank should have two large manholes so placed as to allow of access of fresh air and daylight during the process of cleaning. The filters on most of the liners are made of charcoal, sand, &c., materials long since condemned as worse than useless. For these there should be substituted filters of the Pasteur-Chamberland type, and to ensure their reliable working their periodical cleaning and renewal should be provided for by contract with the makers.

Robinson[9] reviews the mechanical water filters now in common use. They may be divided into four classes: (1) Those of the baked or natural stone tube variety; (2) packed filters of oxidising, or (3) non-oxidising porous powders; and (4) filters using preparatory chemical coagulants. Tube filters of the Berkfeldt or Pasteur-Chamberland varieties have the disadvantage of getting easily clogged, and require every third day to have the tubes scrubbed and boiled, or to have filtered through them a solution of permanganate of potassium, followed by a solution of oxalic acid. Failing such attention, after three or four days bacteria appear on the inside of the tubes. Filters of non-oxidising porous powders, such as sand, flint, &c., are not effective in preventing the passage of bacteria. Oxidising powders, such as charcoal, magnetic carbide of iron, &c., may succeed for a time in keeping back organisms and in oxidising organic matter. This power, however, is soon lost. Of this class the coke filter is the most effective. The addition of coagulants, such as alum, to a water changes the chemical and physical properties of the impurities in such a way as to render filtration more easy; its unpleasant physiological effects, however, forbid its use. A satisfactory filter should consist of three compartments. In the first, after subsidence, the water passes through a bed of fine cracked flint and hardest blue marble; in the second, through magnetic carbide of iron and fine cracked flint; and in the third, through a still finer layer of flint, the interspaces of which should not be larger than those in a Chamberland tube. The preparation of the last-mentioned material is a difficulty which has not yet been overcome.

[1] Tuberculosis as Affecting the Milk Supply; Report to Health Committee. [2] Les Nouveaux Remedes, May 8, 1897. [3] Zeit. für Hygiene, xxii. l. [4] Archiv. für Hygiene, xxvii. 4. [5] Journ. of State Medicine, Jan., 1897. [6] Lancet, Feb. 30, 1897, et seq. [7] "Village Water Supplies," Journ. of State Medicine, Aug., 1897. [8] Brit. Med. Journ., Aug. 7, 1897. [9] American Journal of Medical Sciences, Feb. 1897.

